# Chronic osteomyelitis increases long-term mortality risk in the elderly: a nationwide population-based cohort study

**DOI:** 10.1186/s12877-016-0248-8

**Published:** 2016-03-31

**Authors:** Chien-Cheng Huang, Kang-Ting Tsai, Shih-Feng Weng, Hung-Jung Lin, Hung-Sheng Huang, Jhi-Joung Wang, How-Ran Guo, Chien-Chin Hsu

**Affiliations:** Department of Emergency Medicine, Chi-Mei Medical Center, Tainan, Taiwan, 901 Zhonghua Road, Yongkang District, Tainan City, 710 Taiwan; Bachelor Program of Senior Service, Southern Taiwan University of Science and Technology, Tainan, Taiwan; Department of Environmental and Occupational Health, College of Medicine, National Cheng Kung University, Tainan, Taiwan; Department of Geriatrics and Gerontology, Chi-Mei Medical Center, Tainan, Taiwan; Department of Occupational Medicine, Chi-Mei Medical Center, Tainan, Taiwan; Graduate Institute of Medical Sciences, College of Health Sciences, Chang Jung Christian University, Tainan, Taiwan; Department of Healthcare Administration and Medical Informatics, Kaohsiung Medical University, Kaohsiung, Taiwan; Department of Biotechnology, Southern Taiwan University of Science and Technology, Tainan, Taiwan; Department of Emergency Medicine, Taipei Medical University, Taipei, Taiwan; Department of Medical Research, Chi-Mei Medical Center, Tainan, Taiwan; Department of Occupational and Environmental Medicine, National Cheng Kung University Hospital, Tainan, Taiwan

**Keywords:** Chronic osteomyelitis, Elderly, Long-term mortality

## Abstract

**Background:**

The elderly are predisposed to chronic osteomyelitis because of the immunocompromised nature of aging and increasing number of chronic comorbidities. Chronic osteomyelitis may significantly affect the health of the elderly; however, its impact on long-term mortality remains unclear. We conceived this retrospective nationwide population-based cohort study to address this issue.

**Methods:**

We identified 10,615 elderly patients (≥65 years) comprising 965 patients with chronic osteomyelitis and 9650 without chronic osteomyelitis matched at a ratio of 1:10 by age and gender between 1999 and 2010 from the Taiwan National Health Insurance Research Database. The risk of chronic osteomyelitis between the two cohorts was compared by a following-up until 2011.

**Results:**

Patients with chronic osteomyelitis had a significantly higher mortality risk than those without chronic osteomyelitis [incidence rate ratio (IRR): 2.29; 95 % confidence interval (CI): 2.01–2.59], particularly the old elderly (≥85 years; IRR: 3.27; 95 % CI: 2.22–4.82) and males (IRR: 2.7; 95 % CI: 2.31–3.16). The highest mortality risk was observed in the first month (IRR: 5.01; 95 % CI: 2.02–12.42), and it remained persistently higher even after 6 years (IRR: 1.53; 95 % CI: 1.13–2.06) of follow-up. Cox proportional hazard regression analysis showed that chronic osteomyelitis [adjusted hazard ratio (AHR): 1.89; 95 % CI: 1.66–2.15], advanced age (≥85 years; AHR: 2.02; 95 % CI: 1.70–2.41), male (AHR: 1.34; 95 % CI: 1.22–1.48), and chronic comorbidities were independent predictors of mortality.

**Conclusions:**

This study demonstrated that chronic osteomyelitis significantly increased the long-term mortality risk in the elderly. Therefore, strategies for prevention and treatment of chronic osteomyelitis and concomitant control of chronic comorbidities are very important for the management of the elderly, particularly for a future with an increasingly aged population worldwide.

## Background

The proportion of elderly individuals (≥65 years old) comprised 6.2 % of the world population in 1992, which is expected to rise to 20 % by 2050 [1]. By 2030, 20 % of the U.S. population is estimated to be more than 65 years old [2]. In Taiwan, owing to a decrease in the fertility rate, medical advances, and a comprehensive national healthcare system, the proportion of elderly individuals increased from 7 % in 1993 to 11.33 % in 2013 [3]. This steady increase in the elderly population also necessitates the increase of medical and healthcare resources [4]. In 2013, the cost of medical expenditure for the elderly population was estimated to be 33 % of the total Taiwan National Health Insurance program, which is almost three to four times than that of the non-elderly in terms of the average cost per person [4].

Osteomyelitis is a common musculoskeletal infectious disease in the elderly, second only to skin and soft tissue infection [5]. The elderly are predisposed to osteomyelitis because of the immunocompromised nature of aging [6] and comorbidities such as diabetes mellitus (DM), peripheral vascular disease, pressure ulcers, and surgical interventions [5, 7]. Osteomyelitis can be divided into three subgroups according to the acuteness of the infectious process: acute, subacute, and chronic osteomyelitis (CO) [5]. Osteomyelitis in the elderly is most often caused by pyogenic organisms, followed by *Mycobacterium tuberculosis* [5]. Acute osteomyelitis is usually caused by hematogenous spread, and *Staphylococcus aureus* is the most common pathogen in this context [5, 8]. Antimicrobial therapy alone for 4–6 weeks is usually effective for treating acute and subacute osteomyelitis [5, 9]. CO may be caused by *S. aureus* or Gram-negative organisms such as *Bacteroides fragilis* [5, 9]. Because of the poor blood supply to the infected bone, CO usually requires not only antibiotic therapy but also adequate surgical debridement [5, 6, 9]. Other adjuvant therapies such as antibiotics, hyperbaric oxygen, nutritional supplementation, advice on smoking cessation, tight blood glucose control, arterial bypass surgery, and discontinuation or alteration of medications are recommended according to individual conditions [6]. Despite the treatments, the persistence of CO is not uncommon [5], which results in chronic disability, impairment of the quality of life, and even an increased risk of long-term mortality. Several epidemiological studies have reported that CO increases the risk of subsequent coronary heart disease [10], stroke [11], DM [12], renal disease [13], and depression [14]. However, the long-term mortality risk of CO in the elderly has not yet been clarified. Therefore, we designed a retrospective nationwide population-based cohort study to delineate this issue. We hypothesized that long-term mortality increased in the elderly with CO.

## Methods

### Data sources

**Fig. 1:**
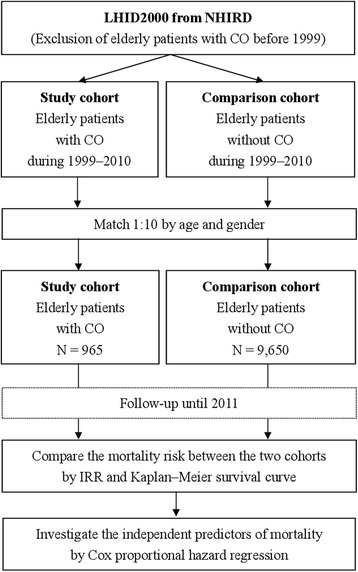
Flowchart of the study. LHID, Longitudinal Health Insurance Database; NHIRD, National Health Insurance Research Database; CO, chronic osteomyelitis; IRR, incidence rate ratio

Taiwan National Health Insurance program was a single-payer program launched on March 1, 1995. Nearly 100 % of Taiwan’s 23.75 million individuals, including foreigners, were enrolled in the program [15]. This study was based on the Longitudinal Health Insurance Database 2000 (LHID2000), which contains all the original claim data of 200,000 individuals randomly sampled from the 2000 Registry for Beneficiaries (ID) of the National Health Insurance Research Database (NHIRD) (Fig. 1) [16]. The NHIRD maintains the registration data of every individual who was a beneficiary of the National Health Insurance program during 1996–2000 [16]. There was no significant difference in gender distribution between the patients in the LHID2000 and the original NHIRD [16].

### Study design, participants, and definitions

Initially, elderly patients (aged ≥ 65 years) who had CO (ICD-9 code: 730.1) before 1999 were excluded (Fig. 1). Then, the elderly patients with CO and those without CO between January 1, 1999, and December 31, 2010, were selected as the study cohort and the comparison cohort, respectively, matched at a ratio of 1:10 by age and gender. We categorized the study population on the basis of their ages into three subgroups: young elderly (65–74 years), moderately elderly (75–84 years), and old elderly (≥85 years) for stratification analysis. CO is usually defined as osteomyelitis with a duration of more than 6 weeks or as recurrent or non-curable osteomyelitis [5]. The comorbidities that affect mortality were also included in the study and defined as follows: DM (ICD-9 code 250), hypertension (HTN) (ICD-9 code 401–405), renal disease (ICD-9 codes 582, 583, 585, 586, 588), stroke (ICD-9 codes 430–438), congestive heart failure (CHF) (ICD-9 code 428), liver disease (ICD-9 codes 5712, 5714, 5715, 5716, 4560–4562, 5722–5728), cancer (ICD-9 codes 140–208), and chronic obstructive pulmonary disease (COPD) (ICD-9 codes 490–496, 500–505, 5064). Monthly income was also included as it is a potential confounding factor. Mortality risk between the two cohorts was compared by following up until 2011. Independent predictors of mortality were also investigated.

### Ethic statements

This study was designed according to the Declaration of Helsinki and approved by the Institutional Review Board at Chi-Mei Medical Center. Informed consent was waived because the LHID2000 used in this study consists of unidentifiable and secondary data released to the public for research purposes [16]. The rights and welfare of the patients were not affected by the waiver.

### Statistical analysis

Age, gender, comorbidities, and monthly income between the two study cohorts were compared using Pearson χ^2^ tests for categorical variables and independent *t* test for continuous variables. Mortality risk was compared by estimating the incidence rate ratio (IRR) with conditional Poisson regression. Kaplan–Meier analysis was used to calculate the cumulative survival rate between the two cohorts. Cox proportional hazard regression analysis was used to investigate the independent predictors of mortality after adjusting for age, gender, DM, HTN, renal disease, stroke, CHF, liver disease, cancer, COPD, and monthly income. SAS 9.3.1 for Windows (SAS Institute, Cary, NC, USA) was used for all statistical analyses. Significance was set at *P* < 0.05 (two-tailed).

## Results

There was no significant difference in age and gender between the two matched cohorts (Table 1). The mean ages of patients with CO and those without CO were 74.89 ± 6.45 and 74.87 ± 6.43 years, respectively. Young elderly (65–74 years) comprised the majority (~55 %) followed by the moderately elderly (75–84 years) (~37 %) in each cohort. There were more male patients with CO than female patients (52 % vs. 48 %). Patients with CO had significantly more number of comorbidities such as DM, HTN, renal disease, stroke, CHF, cancer, and COPD than patients without CO (all *p* < 0.05). There was no significant difference in the monthly income between the two cohorts.

**Table 1:** Demographic characteristics and comorbidities of elderly patients with and without CO

Characteristics	Elderly patients with CO	Elderly patients without CO	*p*-value
	(*N* = 965)	(*N* = 9,650)	
Age (years)	74.89 ± 6.45	74.87 ± 6.43	0.9213
65–74	531 (55.03)	5,322 (55.15)	0.9913
75–84	360 (37.31)	3,599 (37.30)	
≥85	74 (7.67)	729 (7.55)	
Gender			
Male	502 (52.02)	5,020 (52.02)	>0.999
Female	463 (47.98)	4,630 (47.98)	
Comorbidity			
DM	291 (30.16)	1,502 (15.56)	<0.0001
HTN	438 (45.39)	3,369 (34.91)	<0.0001
Renal disease	57 (5.91)	254 (2.63)	<0.0001
Stroke	120 (12.44)	798 (8.27)	<0.0001
CHF	49 (5.08)	238 (2.47)	<0.0001
Liver disease	43 (4.46)	345 (3.58)	0.1645
Cancer	59 (6.11)	364 (3.77)	0.0004
COPD	133 (13.78)	847 (8.78)	<0.0001
Monthly income			
<NT$ 15,840	619 (64.15)	6,252 (64.79)	0.3735
NT$ 15,840–25,000	333 (34.51)	3,213 (33.30)	
>NT$ 25,000	13 (1.35)	185 (1.92)	

Data are N (%) or mean ± standard deviation

CO *chronic osteomyelitis*, *DM* diabetes mellitus, *HTN* hypertension, *CHF* congestive heart failure, *COPD* chronic obstructive pulmonary disease, *NT$* New Taiwan Dollars

**Fig. 2:**
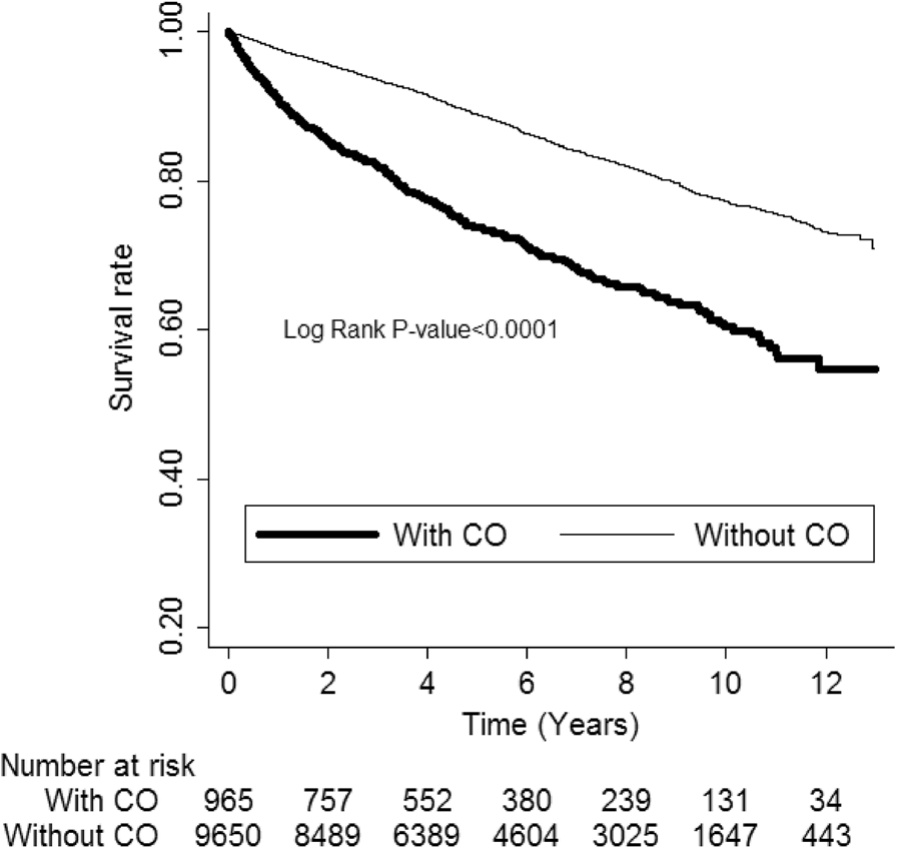
Kaplan–Meier survival analysis of patients with and without CO. CO, chronic osteomyelitis

After the 13-year follow-up period until 2011, patients with CO showed a significantly higher mortality risk than patients without CO [IRR: 2.29; 95 % confidence interval (CI): 2.01–2.59] (Table 2). Stratification analysis of age showed the highest mortality risk of CO in the old elderly (≥85 years) (IRR: 3.27; 95 % CI: 2.22–4.82). Male patients with CO had a higher mortality risk than their female counterparts (IRR: 2.7 vs. 1.77). Subgroup analyses of comorbidities such as DM, HTN, renal disease, stroke, cancer, and COPD also showed a higher risk of mortality in the patients with CO than those without CO. Stratification analysis of the follow-up period data revealed the highest mortality risk in the first month (IRR: 5.01; 95 % CI: 2.02–12.42), and the risk was persistently higher throughout the follow-up period, even after 6 years (IRR: 1.53; 95 % CI: 1.13–2.06), among the patients with CO compared with those without CO. The Kaplan–Meier survival analysis also showed that patients with CO had a significantly lower survival rate than patients without CO (*p* < 0.0001) (Fig. 2).

**Table 2:** Comparison of mortality risk for elderly patients with and without CO

Characteristics	Elderly patients with CO				Elderly patients without CO				IRR (95 % CI)	*p*-value
	N	Death	PY^a^	Rate^b^	N	Death	PY^a^	Rate^b^		
All	965	289	5131.3	56.32	9650	1456	59034.95	24.66	2.29 (2.01–2.59)	<0.0001
Age (years)										
65–74	531	128	3097.15	41.33	5322	643	34007.74	18.91	2.19 (1.81–2.65)	<0.0001
75–84	360	129	1720.28	74.99	3599	688	21018.46	32.73	2.29 (1.9–2.76)	<0.0001
≥85	74	32	313.87	101.95	729	125	4008.75	31.18	3.27 (2.22–4.82)	<0.0001
Gender										
Male	502	194	2611.37	74.29	5020	877	31878.11	27.51	2.7 (2.31–3.16)	<0.0001
Female	463	95	2519.93	37.7	4630	579	27156.85	21.32	1.77 (1.42–2.2)	<0.0001
Comorbidity										
DM	291	113	1446.51	78.12	1502	341	8489.47	40.17	1.94 (1.57–2.41)	<0.0001
HTN	438	134	2286.75	58.6	3369	621	19093.71	32.52	1.8 (1.49–2.17)	<0.0001
Renal disease	57	28	218.35	128.23	254	78	1308.83	59.6	2.18 (1.41–3.36)	0.0004
Stroke	120	52	543.46	95.68	798	215	4255.75	50.52	1.89 (1.4–2.56)	<0.0001
CHF	49	19	208.15	91.28	238	78	1207.95	64.57	1.41 (0.86–2.33)	0.176
Liver disease	43	15	235.48	63.7	345	85	2011.18	42.26	1.51 (0.87–2.61)	0.143
Cancer	59	30	211.4	141.91	364	104	1670.29	62.26	2.28 (1.52–3.42)	<0.0001
COPD	133	52	644.82	80.64	847	256	4937.64	51.85	1.56 (1.15–2.1)	0.0037
Follow-up period										
0–1 month	965	7	80.15	87.34	9650	15	803.5	18.67	5.01 (2.02–12.42)	0.0005
1–6 months	958	43	389.54	110.39	9635	86	3997.24	21.52	5.13 (3.56–7.4)	<0.0001
6–12 months	915	37	448.48	82.5	9549	127	4742.7	26.78	3.08 (2.14–4.44)	<0.0001
1–2 years	878	51	815.21	62.56	9422	188	8964.55	20.97	2.98 (2.19–4.07)	<0.0001
2–4 years	757	64	1305.36	49.03	8489	328	14932.6	21.97	2.23 (1.71–2.92)	<0.0001
4–6 years	552	39	917.42	42.51	6389	320	10909.76	29.33	1.45 (1.04–2.03)	0.0274
>6 years	380	48	1175.14	40.85	4604	392	14684.61	26.69	1.53 (1.13–2.06)	0.0054

^a^ *PY* person-years

^b^Rate: per 1000 person-years

Data are N (%) or mean ± standard deviation

*CO* chronic osteomyelitis, *IRR* incidence rate ratio, *CI* confidence interval, *DM* diabetes mellitus, *HTN* hypertension, *CHF* congestive heart failure, *COPD* chronic obstructive pulmonary disease

Cox proportional hazard regression analysis showed that CO was an independent predictor of mortality after adjusting for age, gender, comorbidities, and monthly income [adjusted hazard ratio (AHR): 1.89; 95 % CI: 1.66–2.15] (Table 3). In addition, advanced age, male gender, and all of the comorbidities included in this study were independent predictors of mortality.

**Table 3:** Cox proportional hazard regression analysis for mortality risk in the elderly patients

Cohort	Crude hazard ratio (95 % CI)	Adjusted hazard ratio (95 % CI)
Chronic osteomyelitis		
Yes	2.29 (2.02–2.59)*	1.89 (1.66–2.15)*
No	1.000	1.000
Age (years)		
65–74	1.000	1.000
75–84	1.74 (1.57–1.91)*	1.69 (1.53–1.87)*
≥85	1.76 (1.49–2.10)*	2.02 (1.70–2.41)*
Gender		
Male	1.36 (1.24–1.50)*	1.34 (1.22–1.48)*
Female	1.000	1.000
Comorbidity		
DM	1.92 (1.73–2.14)*	1.71 (1.53–1.92)*
HTN	1.53 (1.39–1.68)*	1.16 (1.05–1.29)*
Renal disease	2.66 (2.19–3.24)*	2.03 (1.66–2.47)*
Stroke	2.24 (1.97–2.56)*	1.82 (1.59–2.08)*
CHF	2.61 (2.13–3.20)*	1.86 (1.51–2.29)*
Liver disease	1.68 (1.37–2.05)*	1.47 (1.201.81)*
Cancer	2.77 (2.32–3.31)*	2.41 (2.02–2.88)*
COPD	2.25 (1.99–2.54)*	1.77 (1.56–2.01)*
Monthly income		
<NT$ 15,840	1.71 (1.08–2.68)*	1.50 (0.95–2.36)
NT$ 15,840–25,000	1.34 (0.85–2.12)	1.27 (0.8–2.02)
>NT$ 25,000	1.000	1.000

*p-value < 0.05

*CI* confidence interval, *DM* diabetes mellitus, *HTN* hypertension, *CHF* congestive heart failure, *COPD* chronic obstructive pulmonary disease, *NT$* New Taiwan Dollars

## Discussion

This cohort study demonstrated that CO significantly increased the risk of long-term mortality in the elderly patients during the 13-year follow-up period. The effect was highest in the first month, which persisted even after 6 years, and the impact of CO was directly proportional to the age of the patients. Male patients had a higher mortality risk than female patients if they had CO. In addition to CO, comorbidities such as DM, HTN, renal disease, stroke, CHF, liver disease, cancer, and COPD were also independent predictors of mortality.

The increased long-term mortality in the elderly due to CO could probably be attributed to infection, chronic inflammatory reaction, and CO-related chronic complications such as impaired limb function and psychological status. CO can occur as a result of microorganisms originating from the hematogenous route, spread of infection to the bone from the adjacent soft tissues and joints, or post-trauma or post-surgery infection [5, 17]. *S. aureus* is the commonest causative organism of hematogenous CO, although multiple organisms are usually isolated in cases where CO results from direct inoculation or through contiguous spread [5, 18]. Underlying the pathological processes of infection are inflammation, suppuration, necrosis, exudation, vascular congestion and intraosseous HTN, intravascular thrombosis with occlusion of blood flow, and reactive new bone formation [19]. Because of the presence of sequestrum (dead bone) in CO, it is always very difficult to eradicate the infection completely [5, 7, 19]. The mainstay treatments for CO are surgical debridement, management of ensuing dead space, adjunctive antibiotic therapy, reconstruction of soft tissue defects, and hyperbaric oxygen therapy [5, 7, 19]. However, the elderly patients are always too frail to receive complete surgical debridement with adjunctive therapy in the real situation, which results in the non-curable nature of the disease and a vicious cycle of the general conditions.

Chronic inflammatory reaction is a risk factor for cardiovascular disease and even death [20, 21]. Inflammation triggers the production of proinflammatory cytokines in the arterial wall [22]. Primary cytokines such as tumor necrosis factor-α and interleukin-1 mediate the attraction and migration of inflammatory cells into the vascular tissue [22]. They also induce the “messenger” cytokines, which are released into the systemic circulation, causing the liver to increase the production of acute phase reactants such as C-reactive protein and serum amyloid A, which amplify the inflammatory and procoagulant responses [22–24]. Other risk factors including concomitant DM, HTN, and smoking also add to the inflammation [21]. Finally, all these processes lead to vascular atherosclerosis and cardiovascular events.

The high number of comorbidities suggesting a poor underlying condition renders the elderly particularly vulnerable to CO, which in turns causes more chronic complications including limb deformities, limb length inequality, impaired limb function, pathological fractures, malignant transformation, compartment syndrome and Volkmann contracture, coronary heart disease [10], stroke [11], DM [12], chronic renal failure [13], loss of self-esteem and depression [14], and secondary amyloidosis leading to nephrotic syndrome [19]. Our results showed that elderly patients with chronic comorbidities had higher mortality risk when they had CO. Furthermore, chronic comorbidities also predicted the subsequent mortality in the elderly. The extremity disability caused by CO results in decreased daily activities and elevated risk of other chronic physical and psychological disorders [14], which also may increase the risk of mortality when combining with previous comorbidities. This is a vicious circle suggesting that both prevention of CO and control of comorbidities are very important management strategies for the elderly with CO.

There are some limitations to this study. First, there was no detailed information regarding the treatment strategies (e.g., surgery, antibiotics, hyperbaric oxygen, etc.), laboratory data, lifestyle, and personal health factors including smoking and obesity, which may be confounding factors. Second, we did not classify CO into subgroups based on the affected area (i.e., infection site) such as vertebrae, hip, sacrum, sternum, or mandible and the induced mechanisms such as traumatic, hematogenous, diabetic, post-surgery or pressure ulcer, which may have a different prognosis. Nevertheless, as this study was designed to investigate the long-term effects of CO in the elderly, subgroup analysis was beyond the primary goal. Third, potential information biases due to misclassification by the ICD-9-CM diagnosis codes in NHIRD may exist; however, this misclassification is likely nondifferential. Because nearly 100 % Taiwan’s 23.75 million individuals were enrolled into NHIRD, we thought the selection bias is very small. Fourth, we did not have the data of direct causes of death in this study. Further studies are warranted to address these issues. For example, a prospective well-designed study with detailed data including mechanism of infection, infection site, treatment strategies, and more important measures for physical as well as psychological assessment such as SF-36 (Short Form 36 Health Surveys), would better evaluate the effect of CO on mortality in the elder patients.

## Conclusions

This is the first nationwide population-based cohort study delineating that CO increased the long-term mortality in the elderly, particularly in the males and those with an advanced age. The effect was most significant in the first month and persisted even after 6 years. In addition to CO, chronic underlying comorbidities also predict the mortality. Early prevention and treatment of CO and concomitant control of comorbidities are the suggested management strategies for the elderly with CO.

## Availability of data and materials

The dataset supporting the conclusions of this article is included within the article.

## Abbreviations

IRR: incidence rate ratio
CI: confidence interval
AHR: adjusted hazard ratio
DM: diabetes mellitus
CO: chronic osteomyelitis
LHID: Longitudinal Health Insurance Database
NHIRD: National Health Insurance Research Database
ICD: International Classification of Diseases
HTN: hypertension
CHF: congestive heart failure
COPD: chronic obstructive pulmonary disease
